# Dental Preventive Policies and Socio-Economic Inequalities in Oral Health: A Panel Data Analysis of EU Countries During and After COVID-19

**DOI:** 10.3390/healthcare14111479

**Published:** 2026-05-27

**Authors:** Cassandra Lupita, Anca-Cristina Perpelea, Laura-Cristina Rusu, Iulia Muntean, Oana-Ramona Lobonț, Magda-Mihaela Luca

**Affiliations:** 1 Department of Oral Pathology, Multidisciplinary Center for Research, Evaluation, Diagnosis and Therapies in Oral Medicine, “Victor Babes” University of Medicine and Pharmacy Timisoara, 2 Eftimie Murgu Sq., 300041 Timisoara, Romania; cassandra.lupita@umft.ro (C.L.); iulia.sauciur@umft.ro (I.M.); 2 Department of Organization, Professional Legislation and Management of the Dental Office, Faculty of Dental Medicine, “Carol Davila” University of Medicine and Pharmacy, 17-23 Plevnei Street, 020021 Bucharest, Romania; 3 Finance, Business Information Systems and Modeling Department, Faculty of Economics and Business Administration, West University of Timisoara, 300115 Timisoara, Romania; oana.lobont@e-uvt.ro; 4 Department of Pediatric Dentistry, Faculty of Dental Medicine, “Victor Babes” University of Medicine and Pharmacy Timisoara, Eftimie Murgu Square 2, 300041 Timisoara, Romania; luca.magda@umft.ro

**Keywords:** dental preventive policies, oral health inequalities, unmet dental care needs, socio-economic disparities, out-of-pocket payments, European Union, panel data analysis, COVID-19 pandemic

## Abstract

**Highlights:**

**What are the main findings?**

- Higher out-of-pocket expenditure was consistently associated with higher levels of unmet dental care needs across European countries.
- Socio-economic and health system factors, including education, GDP per capita, and dentist density, contributed to cross-country variation in access to dental care.

**What are the implications of the main findings?**

- Reducing direct household payment burden may help improve access to dental care during periods of system stress.
- Preventive dental policies may be more effective when embedded within broader social and financial protection strategies.

**Abstract:**

**Background/Objectives**: Health system socio-economic inequities in dental care are a long-standing problem in Europe. The issue gained increased relevance during the recent pandemic due to service disruption and socio-economic inequities that become even more pronounced under such circumstances. However, while preventive dental programs are considered key elements of public health, little is known about their role in addressing equity in accessing dental care among different countries and over time between them. This research aims at investigating the relationship between preventive dental policy, socio-economic factors, and the inability to get appropriate dental care within EU member states. **Methods**: A longitudinal panel dataset at the country level, consisting of data collected during 2020 through 2024, was assembled using open sources of statistics from Europe and other international statistical databases. The dependent variable used in the study was the percentage of the population that had unmet dental care need because of cost. Independent variables were the presence or absence of preventive policies related to dentistry, educational attainment, gross domestic product per capita, unemployment rate, number of dentists, and out-of-pocket expenses. Balanced panel datasets and regressions with robust standard errors in random-effects models were estimated. Interaction terms were created to test the moderating effect of education level on the relationship between policies and access to care. **Results**: Cross-country variations in terms of the prevention policy environment, socio-economic status, and unmet dental care need were found from descriptive analysis. The higher level of out-of-pocket payment was always related to the higher unmet dental care need, while the lower GDP countries displayed poorer access. Using the balanced panel random-effects model, preventive dental policies and the interaction between preventive policies and educational level were insignificant factors predicting the unmet dental care need. On the other hand, higher out-of-pocket payments, education, and dentists per million population had nearly significant positive relationships. In the sensitivity analysis, GDP per capita showed a negative association, whereas dentists per million population remained positively associated with unmet dental care need. **Conclusions**: The findings suggest that inequalities in access to dental care during and after the COVID-19 period were shaped primarily by financial and structural determinants rather than by the presence of preventive policies alone. While preventive programs remain an important component of long-term oral health strategies, reducing direct household payment burden and strengthening health system capacity may represent more immediate mechanisms for maintaining equitable access to dental services during periods of system disruption.

## 1. Introduction

Oral health has, for a long time, already passed into the core of public health policy and is extending beyond the boundaries of dentistry. Today, untreated dental conditions are not only a clinical problem, but they are increasingly recognized as a marker of how well a health system functions and how fairly it distributes care. Preventive services sit at the center of this discussion. When they work, they keep people out of dental emergency offices, reduce long-term costs, and maintain quality of life. When they fail, or when access to them becomes uneven, the consequences appear most clearly, especially in vulnerable groups. Across Europe, most health systems formally support prevention, yet the real-world availability of preventive dental care still varies considerably between countries and social groups. This gap between policy on paper and access in practice remains one of the defining challenges of modern oral health systems [1,2,3].

The relationship between preventive dental services and access to care can be understood through several established public health and health policy frameworks. The Theory of Access to Healthcare, particularly Andersen’s Behavioral Model, explains how individual characteristics, enabling resources, and system-level factors jointly determine the utilization of health services. Complementing this perspective, the Social Determinants of Health framework highlights the role of income, education, employment status, and living conditions in shaping health behaviors and outcomes across populations. In addition, the Health Equity framework distinguishes between equality and equity in health service provision, emphasizing the need for targeted interventions to reduce structural disparities. From a systems perspective, theories related to health system financing and welfare state organization underline the importance of financial protection mechanisms, service capacity, and policy design in ensuring equitable access to preventive care. Finally, public health prevention theory suggests that consistent preventive interventions can reduce long-term treatment costs and mitigate inequalities by addressing risk factors before disease progression occurs.

Despite steady improvements in oral health across Europe over the past decades, access to dental care remains closely tied to social and economic position. Individuals with lower income, lower levels of education, or unstable employment are consistently more likely to postpone treatment or avoid preventive visits altogether, often for reasons that have little to do with clinical need and much more to do with affordability or availability of services. Even in countries with well-developed health systems, out-of-pocket payments continue to shape patterns of utilization, sometimes subtly, sometimes quite visibly, especially when household budgets are under pressure.

These differences rarely appear overnight. They build gradually, influenced by everyday decisions about whether to seek care now or wait, whether prevention feels affordable or optional, and whether the system feels accessible or distant. Over time, the result is a familiar pattern: populations already facing social disadvantages tend to accumulate a higher burden of untreated dental disease and report more frequent barriers to care. From a policy perspective, this is where prevention becomes more than a clinical strategy. It becomes a mechanism through which health systems can either narrow existing gaps or, unintentionally, allow them to widen depending on how financial protection and service organization are structured [4,5,6].

The COVID-19 pandemic introduced an unexpected stress test for health systems across Europe, and dental services were among the sectors most visibly affected. In many countries, routine consultations were postponed, preventive programs were temporarily suspended, and access to care became more difficult, particularly during the early stages of the pandemic. For several months, the focus of health systems shifted toward urgent and emergency services, leaving preventive care in the background. This disruption was not uniform across countries, nor across population groups. Some systems adapted quickly and restored services within a relatively short time, while others experienced longer interruptions and slower recovery.

From a public health perspective, the pandemic did not create inequalities in access to dental care, but it exposed and, in some cases, amplified those that were already present. Individuals with stable income and reliable insurance coverage were generally able to return to regular care once services resumed. In contrast, those facing financial constraints or living in areas with limited service availability often delayed treatment for longer periods, sometimes until symptoms became unavoidable. This sequence of events turned the year 2020 into a clear turning point, followed by a gradual phase of adjustment and recovery in the years that followed. Understanding how preventive policies functioned during this period offers a useful opportunity to observe how health systems respond when routine access is suddenly disrupted [7,8,9].

There is now abundant literature documenting the positive effects of preventive oral healthcare as a foundation for the promotion of general oral health among populations [10,11,12]. Studies on preventive programs involving early detection and check-ups, as well as community-level prevention initiatives, have indicated low prevalence of untreated conditions and better health outcomes for diverse groups of patients [10,11,12]. Early preventive interventions initiated during childhood and delivered in a school-based context have proven to be efficient in achieving significant health gains, which persist into adulthood, as well as lowering future treatment expenses [13,14]. As a result, several European countries’ healthcare providers have increased their efforts to develop and implement preventive policies.

Against this background, the present study set out to examine how preventive dental policies were associated with changes in access to dental care across European Union countries during the period 2020–2024, a timeframe marked first by disruption and then by gradual recovery of routine health services [15,16]. The analysis focused on the relationship between preventive program implementation, financial barriers to care, and socio-economic characteristics of national populations, with particular attention to how these factors influenced reported unmet dental care needs [15,16]. This study contributes to the existing literature in several important ways. First, it provides a multi-country longitudinal analysis of preventive dental policies and access to care across European Union member states during a period marked by both disruption and recovery [17,18,19]. Second, it integrates preventive policy indicators with key socio-economic determinants, allowing for a more comprehensive assessment of structural factors influencing unmet dental care needs. Third, the study captures temporal dynamics associated with the COVID-19 pandemic, offering insights into how health systems respond to sudden shocks and how inequalities evolve during recovery phases [7,8,9]. By combining comparative policy analysis with longitudinal econometric modeling, the study advances understanding of the role of preventive strategies in promoting equitable access to dental care [20,21,22].

In addition, recent studies have emphasized the need to explore the impact of health policy frameworks in promoting the described trends. It has become apparent that preventive programs are best suited for ensuring equal access to dental care in the case of consistent application and reliable financing, which is facilitated by adequate organizational models [23,24,25]. However, evidence from cross-country analyses suggests that financial protection mechanisms and service capacity may play an equally important role in shaping access to care, even considering the supply of oral healthcare resources.

More recent analyses have focused on the role of national health policies in shaping these patterns, suggesting that preventive strategies are most effective when they are implemented consistently and supported by stable financing and clear organizational structures [23,24,25]. Where preventive programs are fragmented or unevenly distributed, disparities in access tend to persist despite overall improvements in service availability [26,27]. Taken together, this growing body of evidence underscores the importance of examining preventive policies not only in terms of clinical effectiveness but also in relation to their capacity to reduce inequalities in access to care across different social and economic contexts.

However, despite the growing body of research on preventive dentistry and access to dental services, important gaps remain in the existing literature. Most available studies rely on cross-sectional designs, which provide only a static snapshot of access to care and do not capture how inequalities evolve over time. This limitation becomes particularly relevant in the context of systemic disruptions, such as the COVID-19 pandemic, when health systems experience rapid changes in service delivery and recovery patterns. Furthermore, multi-country longitudinal analyses examining the interaction between preventive policies, socio-economic determinants, and financial barriers to care remain relatively scarce. As a result, there is still insufficient evidence on how preventive strategies influence access to dental care during periods of crisis and subsequent system recovery.

The analysis is guided by the following research hypotheses:

**H1:**  *Financial barriers to care, proxied by out-of-pocket health expenditure, are positively associated with higher levels of unmet dental care needs across European Union countries.*

**H2:**  *Preventive dental policies are associated with reduced unmet dental care needs; however, their effectiveness varies depending on socio-economic context, particularly levels of education and national income.*

**H3:**  *Socio-economic indicators, including gross domestic product per capita, education level, and unemployment rate, are significantly associated with variations in unmet dental care needs across countries and over time.*

Rather than looking at single countries in isolation, the study employs a panel data econometric approach to capture both cross-country differences and temporal changes in access to dental care across European Union member states. This methodological framework enables the identification of structural relationships between preventive policies, socio-economic determinants, and financial barriers to care over time.

For clarity, the remainder of the article is organized as follows. The next section describes the study design, data sources, and variables included in the analysis, together with the econometric approach used to examine the relationship between preventive policies and access to dental care. The Results section then presents the main findings, followed by a discussion of their implications for health policy and service organization. The article concludes with a brief summary of the main observations and their relevance for future oral health policies aimed at improving equitable access to dental care.

## 2. Materials and Methods

### 2.1. Study Design

The present study was conducted as a longitudinal observational analysis of country-level data collected from European Union Member States between 2020 and 2024. The aim was to examine the relationship between preventive dental policies, selected socio-economic indicators, and access to dental care during a period characterized by disruption of routine services followed by gradual recovery. The analysis was based on aggregated national data reported by international statistical sources and focused on population-level patterns rather than individual clinical outcomes.

A panel data structure was used to follow changes within countries over time and to compare trends across different health systems during the study period.

### 2.2. Study Setting and Time Frame

The analysis covered the period from 2020 to 2024, using annually reported data from European Union Member States. This timeframe was selected to capture changes in access to dental care during a period marked initially by the disruption of routine services and later by the gradual restoration of preventive and treatment activities.

The year 2020 represented an exceptional context for health systems across Europe, when many countries temporarily reduced or reorganized dental services in response to public health restrictions. In the following years, services were progressively re-established, allowing patterns of utilization and access to stabilize. Observing this sequence of disruption and recovery provided a practical opportunity to assess how preventive policies functioned under changing system conditions.

### 2.3. Data Sources

The analysis was based on data obtained from publicly available international statistical databases commonly used in health and socio-economic research. Information on access to dental care, preventive services, and population characteristics was primarily drawn from the Eurostat database, including indicators related to unmet dental care needs and socio-economic conditions across European Union Member States [28]. Additional data on health system characteristics and financial indicators were retrieved from the Organization for Economic Co-operation and Development (OECD) statistics portal [29] and the World Health Organization Global Health Observatory database [30], ensuring consistency in definitions and reporting standards across countries.

All variables were collected at the national level and reported annually. The datasets used in this study contain aggregated population statistics and do not include individual-level information. Because the data are publicly available and anonymized, no restrictions apply to their use for research or publication purposes. These sources are widely recognized for their methodological rigor and comparability, making them suitable for longitudinal and cross-country evaluations of health system performance.

The selected variables reflect key structural determinants of healthcare access, including financial capacity, service availability, and socio-economic vulnerability. This conceptual framework guided the selection of explanatory variables included in the analysis.

The dependent variable represented the proportion of individuals reporting unmet dental care needs due to cost.

Preventive dental policies were operationalized as an ordinal policy indicator reflecting the level of implementation of national preventive oral health programs. The preventive policy variable (PREVENT) was coded using a three-level scale:

0—No structured national preventive dental program.
1—Partial or regionally implemented preventive initiatives.
2—Comprehensive nationwide preventive policies with systematic population coverage.

Table 1 presents the definitions, measurement units, and sources of all variables used in the analysis.

This classification was based on official national health policy documentation and publicly reported preventive program coverage data compiled from Eurostat and national health authorities.

There were several socio-economic variables. The gross domestic product per capita measured in purchasing power standards (PPS) served as an indicator of economic growth and living conditions. The GDP per capita values were taken from the Eurostat database. Education and unemployment were measured using indicators obtained from Eurostat datasets.

Health system infrastructure and access to healthcare services were measured by the ratio of practicing dentists to population size and the percentage of out-of-pocket health expenditure. Dentist density was selected as a proxy for healthcare service availability because the number of practicing dentists directly affects geographic and functional access to dental services.

Unemployment rate was included as an indicator of socio-economic vulnerability and financial instability. Out-of-pocket expenditure was used as a proxy for financial barriers to healthcare access, as higher direct payments increase the likelihood of unmet healthcare needs.

All downloaded datasets were subsequently processed, harmonized, and analyzed using Python (version 3.14), using standardized procedures to ensure consistency in variable definitions, time coverage, and country identifiers. Data processing and econometric analyses were performed using the pandas, numpy, linearmodels, statsmodels, and scipy libraries in a reproducible computational environment.

Data harmonization included alignment of reporting periods, verification of measurement units, and standardization of country identifiers based on ISO country codes. Extreme values were examined using descriptive statistics and graphical diagnostics. No systematic outliers requiring removal or winsorization were identified; therefore, all observations were retained in the final dataset.

### 2.4. Study Population and Panel Structure

The analysis comprised EU member states for which comparable statistics regarding access to dentistry and socio-economic indicators were collected within the timeframe of the research. Initially, 25 European Union countries were identified as eligible for inclusion based on data availability for the study period.

Countries with missing information for key indicators were excluded to preserve analytical validity and consistency. Specifically, countries were excluded if at least one essential variable required for econometric modeling was missing for the entire study period.

Data were arranged in a country–year dataset with a yearly observation for each country for the period from 2020 to 2024. For the purpose of econometric analysis, a balanced panel dataset was constructed. In this study, a balanced panel refers to a dataset in which each country included in the final analytical sample has observations for all variables across the entire study period. With regard to selected secondary indicators, in case of any gaps in data, the missing values were estimated using a linear interpolation technique.

Linear interpolation was applied only when missing values occurred between two valid observations within the same country time series. This method is widely used in panel data analysis to preserve temporal continuity and avoid unnecessary loss of observations. Overall, fewer than 5% of total observations required interpolation across all variables.

As for the regression analysis that required information for all variables included in the model, a balanced panel dataset was specified. After applying data completeness criteria, the final analytical sample included 21 countries with complete information for all variables included in the regression models. Sensitivity checks were performed to verify that the exclusion of countries with incomplete data did not materially affect the distribution of key variables, reducing the likelihood of selection bias.

Overall, the final data collection framework comprised annual repeated observations for each country. Such an approach enabled the analysis of cross-sectional differences as well as changes over time within countries.

### 2.5. Statistical Analysis

Descriptive statistics were then calculated in order to describe the distribution of all variables included in the analysis. Descriptive statistics including means, standard deviation, minimum and maximum values were calculated for each variable for all countries and years.

To examine the relationship between socio-economic conditions and access to dental services, panel data regression models were employed. The panel data structure of the data provided the possibility to analyze the effect both between countries and within one particular country over time.

Both fixed-effects and random-effects regression models were estimated to explore possible relationships. While fixed-effects regression models controlled for unobserved heterogeneity that may be present due to country-specific characteristics not changing over time, random-effects models took into account both country-specific unobserved characteristics and variations in observations made in different countries. The choice of the appropriate model specification was determined using the Hausman test. In addition, several diagnostic tests were conducted to evaluate the validity of the regression assumptions. Multicollinearity among independent variables was tested by calculating variance inflation factors (VIFs). The threshold of 5 was set to detect possible multicollinearity problems. Heteroskedasticity was assessed using the Breusch–Pagan test, and serial correlation in panel data was evaluated using the Wooldridge test. Cross-sectional dependence across countries was examined using the Pesaran CD test.

The main regression analysis was conducted using a balanced panel specification including only those countries with complete information for all explanatory variables.

Robust standard errors were estimated using standard errors clustered at the country level to account for heteroskedasticity and serial correlation within countries over time. Statistical significance was evaluated using a conventional threshold of *p* < 0.05. All statistical analyses were conducted using Python (version 3.14) in a reproducible computational environment. Data management and econometric analyses were performed using the pandas and numpy libraries, while panel regression models were estimated using the linearmodels package. Statistical diagnostic tests were conducted using statsmodels and scipy.

### 2.6. Econometric Model

To evaluate the relationship between preventive dental policies and inequalities in access to dental care, panel data regression models were specified using country-level observations collected over the study period. The analysis focused on unmet dental care needs as the primary outcome, reflecting barriers to accessing services due to financial or structural constraints.

The general econometric specification used in the study can be expressed as follows:

(1) $$UnmetNeeds_{it}=\alpha +\beta Preventive_{it}+\delta (Preventive_{it}\times Education_{it})+\gamma X_{it}+\varepsilon_{it}$$

where *i* denote the country and t denotes the year. The dependent variable (UnmetNeeds) represents the proportion of individuals reporting unmet dental care needs due to cost. The variable Preventive reflects the presence of organized preventive dental programs at the national level.

The full econometric specification for the fixed-effects model can be expressed as follows:

(2) $$UnmetNeeds_{it}=\alpha +\beta Preventive_{it}+\delta \left(Preventive_{it}\times Education_{it}\right)+\gamma X_{it}+u_{i}+\lambda_{t}+\varepsilon_{it}$$

where $u_{i}$ represents unobserved country-specific effects and $\lambda_{t}$ represents year-specific effects capturing common temporal shocks affecting all countries simultaneously.

For comparison purposes, the random-effects specification can be expressed as follows:

(3) $$UnmetNeeds_{it}=\alpha +\beta Preventive_{it}+\delta \left(Preventive_{it}\times Education_{it}\right)+\gamma X_{it}+u_{i}+\varepsilon_{it}$$

where $u_{i}$ denotes the random country-specific effect assumed to be uncorrelated with the explanatory variables.

An interaction term between preventive policies and education level was included in the model to examine whether the impact of preventive programs differed across socio-economic contexts. In this regard, the model provides for an estimation of not only the net impact of the policies in question but also an examination of whether these policies have more impact in societies that differ by education level.

The control variables used in vector X refer to economic, health system, and financial factors. The inclusion of these variables serves the purpose of reducing confounding factors and thus strengthening the estimate. Country and year fixed effects were included simultaneously in the regression models (two-way fixed-effects specification) to control for time-invariant national characteristics and common temporal shocks affecting all countries during the study period.

These shocks included system-wide disruptions associated with the COVID-19 pandemic, policy responses, and macroeconomic changes affecting healthcare access across the European Union. Fixed-effects models control for time-invariant unobserved heterogeneity potentially correlated with explanatory variables, whereas random-effects models assume that unobserved country-specific effects are uncorrelated with the explanatory variables.

Both fixed-effects and random-effects models were estimated, and the final model specification was selected using the Hausman test, which evaluates whether the random-effects estimator is consistent relative to the fixed-effects estimator. Several diagnostic tests were conducted to evaluate the validity of panel regression assumptions. Heteroskedasticity was tested using the Breusch–Pagan test. Serial correlation in panel data was assessed using the Wooldridge test. The Hausman test results indicated that the random-effects specification was appropriate for the final model estimation.

Cross-sectional dependence across countries was evaluated using the Pesaran CD test, which is particularly relevant for multi-country panel datasets such as those involving European Union Member States. The regression models included an intercept term and yearly fixed effects. A separate linear time trend variable was not included because yearly fixed effects fully capture temporal variation across the study period.

To assess the robustness of the findings to pandemic-related disruptions, a sensitivity analysis was conducted by re-estimating the regression models after excluding the year 2020, which represented the peak period of service disruption during the COVID-19 pandemic. The direction and statistical significance of the estimated coefficients remained consistent across all model specifications, indicating that the main results were not driven solely by pandemic-related shocks.

### 2.7. Ethical Considerations

This study was based exclusively on publicly available aggregated data obtained from international statistical databases. No individual-level data were used, and no personal or identifiable information was processed during the analysis.

Because the research relied solely on secondary anonymized data sources, formal ethical approval and informed consent were not required. The study was conducted in accordance with standard principles for responsible use of publicly available statistical information.

## 3. Results

### 3.1. Descriptive Statistics

Descriptive statistics for the main study variables are presented in Table 2, summarizing the distribution of preventive policies, socio-economic indicators, and health system characteristics across 25 European Union countries during the period 2020–2024. The table provides an overview of the variability in access to dental care and related structural factors within the study sample.

The variation in the number of observations across variables reflects differences in data availability across countries and years. Missing values were present for selected health system indicators, particularly dentist density and out-of-pocket expenditure, due to incomplete reporting in certain national statistical systems. To preserve the longitudinal structure of the dataset, missing observations were handled using linear interpolation within countries where adjacent year data were available.

Interpolation was applied only to explanatory variables and did not affect the dependent variable. Overall, fewer than 5% of the total observations required interpolation, indicating that missing data had a limited impact on the structure of the dataset. As a result, the balanced panel used in the regression analysis includes only country–year observations with complete information for the variables included in the econometric models.

Across the observation period, the presence of organized preventive dental programs varied between countries, with an average preventive policy score slightly above one, indicating that many countries had at least one structured prevention initiative in place. However, the range observed in the data reflects substantial heterogeneity in the extent and organization of preventive services across national health systems.

Economic indicators also showed considerable variation. Gross domestic product per capita displayed wide differences between countries, illustrating persistent disparities in economic development within the European Union. Similarly, education levels varied across the sample, suggesting differences in population-level social and health-related resources that may influence patterns of health service utilization.

Health system characteristics followed a comparable pattern of variability. Dentist density differed substantially between countries, reflecting differences in workforce capacity and service availability. Out-of-pocket payments for health services also showed notable variation, indicating differences in financial protection mechanisms and the degree to which individuals are required to contribute directly to the cost of care.

Taken together, the descriptive statistics indicate that European health systems operate under diverse economic, social, and organizational conditions. This variation provides an appropriate context for subsequent econometric analysis aimed at examining how preventive policies and socio-economic factors are associated with differences in access to dental care across countries and over time.

**Figure 1:**
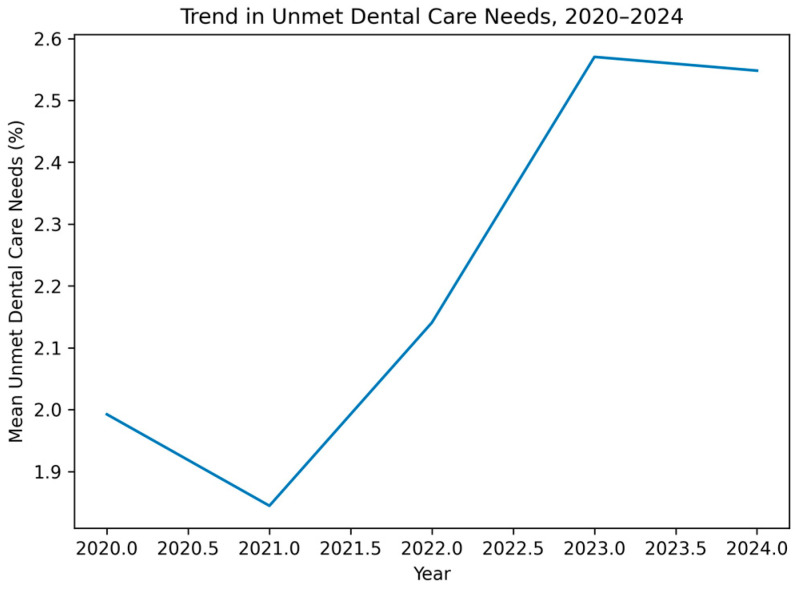
Mean proportion of individuals reporting unmet dental care needs due to cost across European Union countries during the study period (2020–2024). Values represent yearly averages calculated from national-level data.

As shown in Figure 1, the mean level of unmet dental care needs across European Union countries changed over the study period. After a slight decrease between 2020 and 2021, the indicator increased in 2022 and reached its highest level in 2023, with no substantial change observed in 2024. These results indicate a non-linear evolution of unmet dental care needs during the observation period.

**Figure 2:**
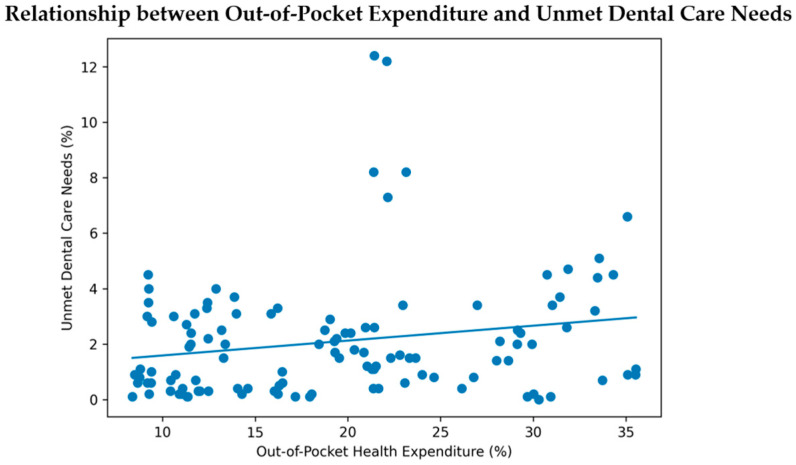
Scatter plot illustrating the relationship between out-of-pocket health expenditure and unmet dental care needs across European Union countries during the study period. Each point represents a country-year observation. The fitted regression line indicates the overall direction of association.

Figure 2 presents the relationship between out-of-pocket health expenditure and unmet dental care needs across European Union countries. The scatter plot shows a positive association between the two variables, with higher levels of direct household payments corresponding to higher levels of unmet dental care needs. Considerable dispersion of observations is visible, reflecting heterogeneity between countries.

### 3.2. Inequality Trends

To further examine inequality patterns in access to dental care, countries were grouped according to structural indicators reflecting economic capacity, financial burden and health system characteristics. Group-based comparisons were used to explore whether unmet dental care needs evolved differently across broader health system contexts over time. Countries were classified into higher and lower groups based on the sample median value of each structural indicator (median split approach). Specifically, countries with values above the median were categorized as the higher group, while those below the median were categorized as the lower group.

**Figure 3:**
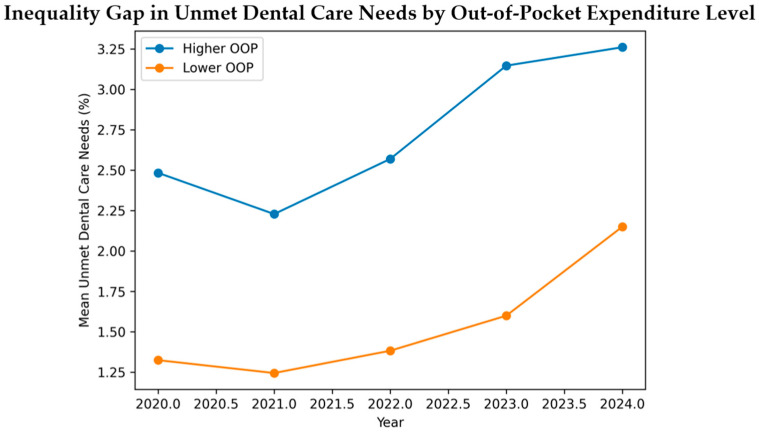
Inequality gap in unmet dental care needs between countries with higher and lower levels of out-of-pocket health expenditure, European Union, 2020–2024. Values represent yearly group averages calculated from national-level data.

Figure 3 presents the yearly average levels of unmet dental care needs in countries with higher and lower levels of out-of-pocket health expenditure. Throughout the study period, countries with higher levels of out-of-pocket health expenditure consistently reported higher average levels of unmet dental care needs than countries with lower levels of out-of-pocket health expenditure. Although both groups showed some variation over time, the gap between them remained visible across the full observation period.

**Figure 4:**
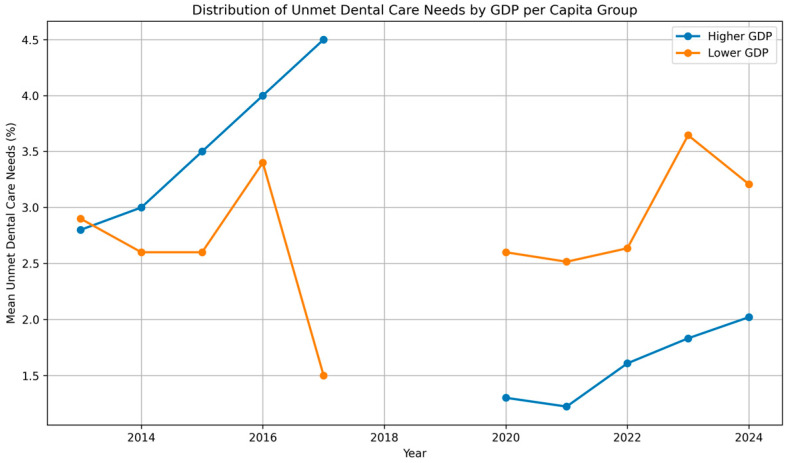
Distribution of unmet dental care needs by country-level GDP per capita group, European Union, 2020–2024. Countries were categorized into higher and lower GDP groups based on the sample median GDP per capita.

A second grouping strategy was based on country-level GDP per capita. As shown in Figure 4, the distribution of unmet dental care needs differed between countries with higher and lower levels of economic development. At the beginning of the observation period, differences between GDP groups were present but not yet consistently defined. From 2020 onward, however, countries in the lower GDP group tended to report consistently higher levels of unmet dental care needs, while countries in the higher GDP group remained at lower average levels.

The visual pattern observed in Figure 4 suggests a clearer differentiation between GDP groups after the initial pandemic year, indicating that economic capacity became a more visible determinant of access to dental care during the period of disruption and recovery. The validity of the difference-in-differences (DiD) approach relies on the parallel trends assumption, which implies that, in the absence of the pandemic shock, trends in unmet dental care needs would have evolved similarly across GDP groups. Visual inspection of pre-pandemic trends suggested broadly comparable trajectories between higher- and lower-GDP countries prior to 2020.

This pre–post contrast provided the basis for an additional difference-in-differences specification, reported in the following section, to assess whether lower-GDP countries experienced a disproportionate increase in unmet dental care needs after 2020.

### 3.3. Difference-in-Differences Analysis

A difference-in-differences specification was estimated to assess whether countries with lower GDP per capita experienced a disproportionate increase in unmet dental care needs following the COVID-19 period after 2020. The model included indicators for lower GDP group membership, the post-2020 period, and their interaction term. The validity of the difference-in-differences approach relies on the parallel trend assumption, which implies that, in the absence of the shock, trends in unmet dental care needs would have evolved similarly across GDP groups. Visual inspection of pre-2020 trends suggested broadly comparable trajectories between groups prior to the pandemic period.

The estimated coefficients from the difference-in-differences model are presented in Table 3. The table summarizes the effects associated with group membership, the post-2020 period, and the interaction term capturing the differential change in unmet dental care needs between GDP groups over time.

The results indicate a strong and statistically significant structural difference between GDP groups. Countries in the lower-GDP category reported substantially higher levels of unmet dental care needs compared to higher-income countries (β = 2.38, *p* < 0.001), confirming the presence of persistent socio-economic inequalities in access to dental services.

However, the interaction term representing the difference-in-differences effect was not statistically significant (β = −1.08, *p* = 0.113), suggesting that the magnitude of inequality between GDP groups did not change significantly after 2020. These findings suggest that disparities in access to dental care were already established prior to 2020 and remained relatively stable throughout the observation period, rather than showing a statistically detectable divergence between GDP groups following the initial disruption.

However, robustness checks commonly used in difference-in-differences analyses, such as placebo tests or event study specifications, were not performed in the present study due to the limited time horizon and the relatively small number of pre-intervention observations. This limitation should be considered when interpreting the causal implications of the estimated difference-in-differences effects.

### 3.4. Panel Regression Results

Panel data regression models were estimated to examine the relationship between preventive dental policies, socio-economic conditions, and access to dental care across European Union countries. The analysis was conducted using a balanced country–year dataset covering the period 2020–2024. Missing observations for selected variables were addressed using linear interpolation to preserve temporal continuity within countries and to ensure comparability across model specifications.

Both fixed-effects and random-effects models were initially estimated as alternative specifications. The selection of the final model specification was guided by the Hausman test, which indicated no statistically significant difference between estimators. Accordingly, the random-effects specification with robust standard errors was retained as the primary model for interpretation.

Differences observed between fixed-effects and random-effects estimates suggest potential sensitivity to unobserved heterogeneity across countries; therefore, both specifications are presented as complementary robustness checks.

The overall model diagnostics and goodness-of-fit indicators are presented in Table 4. The model was estimated using 105 country–year observations from 21 European Union countries observed across five years. The within-country R-squared value (0.1048) indicates modest explanatory power, which is typical for cross-country health system analyses based on aggregated policy and socio-economic indicators.

The estimated regression coefficients are reported in Table 4. Preventive dental policies showed a positive but statistically non-significant association with unmet dental care needs (β = 1.09, *p* = 0.537). The interaction term between preventive policies and education level was not statistically significant (β = −0.002, *p* = 0.928), suggesting that the relationship between preventive policies and access to dental care did not vary systematically across socio-economic contexts.

Education level (β = 0.039, *p* = 0.097), dentist density (β = 0.029, *p* = 0.107), and out-of-pocket expenditure (β = 0.096, *p* = 0.092) demonstrated positive associations with unmet dental care needs, approaching conventional levels of statistical significance. These findings suggest that financial and structural factors may play a more consistent role in shaping access to dental care than preventive policy presence alone.

Potential endogeneity between socio-economic conditions and health outcomes cannot be fully excluded in observational panel data; therefore, the reported associations should be interpreted as indicative rather than strictly causal. Although the inclusion of multiple control variables and country-specific effects reduces the likelihood of omitted variable bias, causal interpretations should be approached with caution (Table 5).

Multicollinearity diagnostics are reported in Table 6. All variance inflation factor (VIF) values were below the commonly used threshold of 5, indicating no evidence of problematic multicollinearity among explanatory variables and supporting the stability of the estimated regression coefficients.

### 3.5. Robustness and Sensitivity Analyses

To assess the robustness of the estimated associations, additional sensitivity analyses were conducted using alternative model specifications. In particular, fixed-effects models with cluster-robust standard errors were estimated to account for potential heteroskedasticity and within-country correlation over time.

The results of the fixed-effects specification are presented in Table 7. This model focuses on within-country variation across the study period and provides a complementary perspective to the random-effects estimates reported in Section 3.4.

In the fixed-effects specification, GDP per capita (β = −0.051, *p* = 0.021) and dentist density (β = 0.051, *p* = 0.049) showed statistically significant associations with unmet dental care needs, indicating that changes in economic conditions and health system capacity within countries may influence access to dental care over time. Education level demonstrated a positive association approaching statistical significance (β = 0.040, *p* = 0.069), while unemployment rate and out-of-pocket expenditure were not statistically significant predictors in this specification.

A comparison between fixed-effects and random-effects specifications indicates a broadly consistent direction of estimated relationships across models. In both specifications, GDP per capita retained a negative association with unmet dental care needs (β = −0.005 in the random-effects model versus β = −0.051 in the fixed-effects model), while education level remained positively associated (β = 0.039 versus β = 0.040), and unemployment rate maintained a negative sign (β = −0.060 versus β = −0.058). These results suggest stability in the direction of associations across model specifications.

However, differences in coefficient magnitude were observed for selected health system indicators. In particular, dentist density showed a stronger effect in the fixed-effects model (β = 0.051) compared to the random-effects specification (β = 0.029), and out-of-pocket expenditure also demonstrated a larger coefficient in the fixed-effects model (β = 0.118) relative to the random-effects model (β = 0.096).

These differences suggest that time-invariant unobserved heterogeneity at the country level may partially influence the estimated coefficients, supporting the interpretation of fixed-effects and random-effects models as complementary rather than competing specifications. Overall, the results are qualitatively robust across alternative panel models, as the direction of key relationships remains stable, although variation in coefficient magnitude indicates moderate sensitivity to model choice.

## 4. Discussion

The current investigation examined the relationship between dental prevention policies, socio-economic factors, and unmet dental care demands among European Union member states at a time when major system upheaval was followed by steady stabilization. The findings suggest that inequality in dental care access is largely associated with structural and economic determinants rather than the mere existence of preventive schemes. While such an outcome might not come as a surprise from a health systems point of view, it still deserves empirical validation. In Europe, oral health services remain more heavily dependent on out-of-pocket costs than other areas of healthcare, and thus, financial barriers play a dominant role in accessing and maintaining dental services [31,32,33].

The stability of the relationship between personal expenses and unmet dental care demands in the current study is consistent with the existing literature on financial determinants of oral healthcare utilization. International studies of the topic have consistently reported that cash payment models have strong links to delayed or avoided dental care, especially in socio-economically deprived populations [34,35]. This structural dependence on private financing distinguishes dental care from most other medical services and helps explain why inequalities in oral health access persist even in countries with otherwise well-developed health systems.

On the contrary, the lack of statistical significance in the relationship between preventive policies and unmet dental care needs should be treated with nuance rather than be considered to contradict well-known preventive theory. A plethora of scientific evidence proves the effectiveness of preventive measures in reducing oral disease risk, whether they be provided in schools or communities or through organized recalls [36]. At the same time, according to the policy-level literature, preventive actions, if practiced without changes in financial and organizational barriers, do not suffice to eradicate inequalities in service utilization [37].

The current analysis, performed against the backdrop of the ongoing pandemic, is especially interesting to interpret. Thus, the temporary increase in the rate of unmet needs in dental care recorded in 2020 and their subsequent normalization in the following years are corroborated by the international experience of the widespread cessation of dental services amid the first months of the coronavirus pandemic [38]. The crucial point is that persisting cross-country differences despite the initial perturbation suggest that the pandemic did not create inequalities but rather suggests pre-existing structural differences in access to dental care.

Another factor that merits particular attention pertains to the significance of structural factors, including economic indicators and labor availability. Based on the current investigation, it was observed that variations in economic performance were associated with variations in unmet needs for dental care in the studied population; this finding is aligned with previous studies indicating a positive relation between macroeconomic development and healthcare access improvement [39,40,41]. However, contrary to what might seem intuitive, an increasing dentist density should not be considered proof that having more providers adversely impacts the studied issue. It merely means that even though there is enough labor available, its allocation cannot be guaranteed, particularly due to the fact that in Europe, dentistry remains urbanized and provided privately rather than publicly. Thus, accessibility of services will continue to suffer even with labor availability.

Overall, the most essential policy implication arising from the current findings involves the realization that preventive dental programs alone cannot solve the problem at hand. The results indicate that, similarly to the situation with other healthcare issues, the success of preventive measures depends on the wider context of their implementation, including financing and access-related aspects. This interpretation corroborates contemporary guidelines that promote oral health integration within universal health coverage frameworks and the reduction in financial barriers as key strategies for improving population oral health outcomes [42].

It is important to discuss some limitations of the current research. First of all, the use of the aggregated national-level dataset limited the identification of personal factors affecting the use of healthcare, including income, health insurance coverage, and accessibility of services within specific geographical areas. Second, the period of observations could be too limited to identify a potential effect since its focus was placed on the situation during the pandemic and the first few years after it ended. Finally, the choice of the preventive policy factor can be associated with its simplification since countries have different levels of implementation and effectiveness of related programs.

To provide a structured interpretation of the empirical findings, the study hypotheses were evaluated against the results obtained from the panel regression and robustness analyses. The comparison between expected theoretical relationships and observed empirical evidence is summarized in Table 8.

The evaluation of hypotheses indicates that financial barriers remain an important structural factor associated with unmet dental care needs, although the strength of this relationship varies across model specifications. Preventive dental policies did not demonstrate a statistically significant moderating effect within the observed period, suggesting that their impact may depend on broader institutional and financial conditions. Socio-economic indicators showed directionally consistent relationships with access to care, but their statistical significance was sensitive to model specification and temporal variation.

Nonetheless, the study can be viewed as an addition to the current literature since it presents a longitudinal approach toward assessing inequalities in access to dental care in the conditions of unprecedented pressure on healthcare systems. In particular, the results of this study highlight the need for financial protection measures, proper organization of the workforce, and overall economic stability in ensuring equity of service access. Thus, future strategies designed to address problems related to unmet dental care needs in Europe should involve investments in preventive programs and structural reforms. Prevention remains a cornerstone of oral health policy, but its full impact can only be realized when supported by a health system capable of delivering services consistently, equitably, and without excessive financial burden for patients.

## 5. Conclusions

This research study supports the assumption that differences in the availability of dental services in various European countries are associated with structural and financial factors rather than to the existence of preventive dental policies.

The empirical findings indicate that preventive policies were not statistically significant predictors of unmet dental care needs in the main panel model; however, this result should not be interpreted as evidence of no impact. Rather, it suggests that preventive measures alone may be insufficient to reduce inequalities in access to care when structural and financial barriers remain present.

The results obtained from panel regression analysis, difference-in-differences estimation, and robustness checks using alternative model specifications indicate broadly consistent patterns across analytical approaches. In particular, financial and structural indicators showed directionally stable relationships with unmet dental care needs, although some sensitivity to model choice was observed between fixed-effects and random-effects specifications.

These findings indicate that improvements in access to dental care are likely to depend on coordinated investments in financial protection mechanisms, workforce organization, and service delivery capacity, alongside sustained preventive actions. In light of recent system disruptions, strengthening the resilience of dental care systems should become a priority for public health policy and health system planning.

Overall, the study contributes to the evidence base on oral health inequalities by providing a longitudinal, cross-country assessment of access to dental care during a period of system disruption and recovery. The findings highlight the importance of integrating preventive programs with broader health system reforms aimed at reducing financial barriers and improving service accessibility.

For future research, it would be important to extend the observation period and incorporate more detailed indicators reflecting the implementation, intensity, and effectiveness of preventive dental policies, as well as individual-level determinants of service utilization.

## Figures and Tables

**Table 1:** Variables description and data sources.

Variable	Abbreviation	Definition	Unit	Source	Type of Variable
Unmet dental care needs	UNMET	Percentage of individuals reporting unmet dental care needs due to cost	% of population	Eurostat—EU Statistics on Income and Living Conditions (EU-SILC)	Dependent (continuous)
Preventive dental policy	PREVENT	Level of implementation of national preventive dental programs	Index (0–2)	Eurostat and national health policy documentation	Independent (ordinal)
GDP per capita	GDP	Economic output per capita adjusted for purchasing power standards	PPS index	Eurostat	Independent (continuous)
Education level	EDUC	Share of population with tertiary education	% of population	Eurostat	Independent (continuous)
Unemployment rate	UNEMP	Percentage of unemployed individuals in the labor force	% of labor force	Eurostat	Independent (continuous)
Dentist density	DENT	Number of practicing dentists relative to population size	Dentists per 100,000 inhabitants	OECD Health Statistics	Independent (continuous)
Out-of-pocket health expenditure	OOP	Share of healthcare costs paid directly by households	% of total health expenditure	OECD Health Statistics	Independent (continuous)

**Table 2:** Descriptive statistics of the main study variables across 25 European Union countries, 2020–2024.

Variable	Mean	Std. Dev.	Min	Max	Observations
preventive	1.12	0.60	0	2	120
gdp	103.73	42.94	57	263	135
education	78.76	14.51	15.6	95.6	135
unemployment	5.85	2.24	2.20	15.5	135
dentists	81.13	20.83	44.7	132.19	83
oop	19.40	8.06	8.39	35.53	115

**Table 3:** Difference-in-differences estimates for unmet dental care needs.

Variable	Coefficient	Std. Error	*p*-Value
Intercept	1.21	0.15	<0.001
Post-2020 period	0.29	0.26	0.257
Lower GDP group	2.38	0.54	<0.001
Post-2020 × Lower GDP	−1.08	0.68	0.113

**Table 4:** Random-effects model diagnostics and goodness-of-fit indicators for unmet dental care needs across European Union countries, balanced panel dataset, 2020–2024.

Indicator	Value
Dependent variable	Unmet dental care needs
Estimator	Random Effects
Covariance estimator	Robust
Number of observations	105
Number of countries	21
Number of years	5
Average observations per country	5
R-squared (Within)	0.1048
R-squared (Between)	0.0118
R-squared (Overall)	0.0207
F-statistic (robust)	1.8867
*p*-value (F-statistic)	0.0799
Log-likelihood	−114.76

**Table 5:** Random-effects panel regression estimates for the association between preventive dental policies, socio-economic indicators, and unmet dental care needs in European Union countries, 2020–2024.

Variable	Coefficient	Std. Error	t-Statistic	*p*-Value
Preventive programs	1.090	1.761	0.619	0.537
Education level	0.039	0.023	1.679	0.097
Preventive × Education	−0.002	0.020	−0.091	0.928
GDP per capita	−0.005	0.012	−0.401	0.689
Unemployment rate	−0.060	0.086	−0.707	0.482
Dentist density	0.029	0.018	1.628	0.107
Out-of-pocket expenditure	0.096	0.056	1.702	0.092
Constant	−5.489	3.699	−1.484	0.141

**Table 6:** Variance inflation factor (VIF) diagnostics for explanatory variables included in the random-effects panel regression model.

Variable	VIF
Preventive programs	2.07
GDP per capita	1.84
Education level	1.36
Unemployment rate	1.06
Dentist density	2.61
Out-of-pocket expenditure	1.57

**Table 7:** Fixed-effects panel regression results with cluster-robust standard errors for unmet dental care needs across European Union countries, 2020–2024.

Variable	Coefficient	Std. Error	*p*-Value
GDP per capita	−0.051	0.021	0.021
Education level	0.040	0.022	0.069
Unemployment rate	−0.058	0.155	0.711
Dentist density	0.051	0.025	0.049
Out-of-pocket expenditure	0.118	0.103	0.258

**Table 8:** Evaluation of research hypotheses.

Hypothesis	Statement	Expected Relationship	Empirical Evidence	Conclusion
H1	Financial barriers (out-of-pocket expenditure) are positively associated with unmet dental care needs	Positive	Positive but not statistically significant in RE; positive and significant in FE model	Partially supported
H2	The effect of preventive dental policies is moderated by socio-economic context (education, income)	Conditional/interaction effect	Interaction term (Preventive × Education) is not statistically significant	Not supported (no robust evidence of moderation)
H3	Socio-economic conditions (GDP, education, unemployment) are associated with unmet dental care needs	Mixed (directional associations expected)	Mixed results: GDP and dentist density significant in FE; weak/non-significant in RE	Partially supported

## Data Availability

The datasets analyzed during the current study are publicly available from international statistical databases, including Eurostat, the Organization for Economic Co-operation and Development (OECD), and related public health data repositories. The processed dataset and statistical code used for analysis are available from the corresponding author upon reasonable request.

## Abbreviations

The following abbreviations are used in this manuscript:

EU	European Union
GDP	Gross Domestic Product
OECD	Organization for Economic Co-operation and Development
OOP	Out-of-pocket expenditure
VIF	Variance Inflation Factor
FE	Fixed Effects
RE	Random Effects
UHC	Universal Health Coverage
COVID-19	Coronavirus Disease 2019

## References

[1] Watt R.G., Daly B., Allison P., Macpherson L.M.D., Venturelli R., Listl S., Weyant R.J., Mathur M.R., Guarnizo-Herreño C.C., Celeste R.K. (2019). Ending the neglect of global oral health: Time for radical action. Lancet.

[2] Peres M.A., Macpherson L.M.D., Weyant R.J., Daly B., Venturelli R., Mathur M.R., Listl S., Celeste R.K., Guarnizo-Herreño C.C., Kearns C. (2019). Oral diseases: A global public health challenge. Lancet.

[3] World Health Organization (2022). Global Oral Health Status Report: Towards Universal Health Coverage for Oral Health by 2030.

[4] Petersen P.E., Kwan S. (2010). The 7th WHO Global Conference on Health Promotion—Towards Integration of Oral Health. Community Dent. Health.

[5] Sanders A.E., Slade G.D., Turrell G., Spencer A.J., Marcenes W. (2006). The shape of the socioeconomic-oral health gradient: Implications for theoretical explanations. Community Dent. Oral Epidemiol..

[6] Sabbah W., Tsakos G., Chandola T., Sheiham A., Watt R.G. (2007). Social gradients in oral and general health. J. Dent. Res..

[7] Guo H., Zhou Y., Liu X., Tan J. (2020). The impact of the COVID-19 epidemic on the utilization of emergency dental services. J. Dent. Sci..

[8] Coulthard P. (2020). Dentistry and coronavirus (COVID-19)—Moral decision-making. Br. Dent. J..

[9] Schwendicke F., Krois J., Gomez J. (2020). Impact of SARS-CoV-2 (COVID-19) on dental practices: Economic analysis. J. Dent..

[10] Fejerskov O., Nyvad B., Kidd E. (2015). Dental Caries: The Disease and Its Clinical Management.

[11] Marinho V.C.C., Worthington H.V., Walsh T., Clarkson J.E. (2013). Fluoride varnishes for preventing dental caries in children and adolescents. Cochrane Database Syst. Rev..

[12] Twetman S. (2018). Prevention of dental caries as a non-communicable disease. Eur. J. Oral Sci..

[13] Jackson S.L., Vann W.F., Kotch J.B., Pahel B.T., Lee J.Y. (2011). Impact of poor oral health on children’s school attendance. Am. J. Public Health.

[14] Griffin S.O., Jones K., Tomar S.L. (2001). An economic evaluation of community water fluoridation. J. Public Health Dent..

[15] Watt R.G., Sheiham A. (2012). Integrating the common risk factor approach into a social determinants framework. Community Dent. Oral Epidemiol..

[16] Petersen P.E. (2003). Improvement of oral health in the 21st century: The approach of the WHO Global Oral Health Programme. Community Dent. Oral Epidemiol..

[17] Listl S., Galloway J., Mossey P.A., Marcenes W. (2015). Global economic impact of dental diseases. J. Dent. Res..

[18] Birch S., Listl S. (2015). The economics of oral health and dental care. J. Dent. Res..

[19] Petersen P.E., Ogawa H. (2016). Prevention of dental caries through the use of fluoride—The WHO approach. Community Dent. Health.

[20] Birch S., Bridgman C. (2010). Increasing access to dental care: Policy implications. Health Policy.

[21] Manski R.J., Magder L.S. (1998). Demographic and socioeconomic predictors of dental service utilization. J. Public Health Dent..

[22] Guarnizo-Herreño C.C., Watt R.G. (2011). Equity, social determinants and public health programmes—The case of oral health. Community Dent. Oral Epidemiol..

[23] Vernazza C., Birch S., Pitts N. (2021). Reorienting oral health services to prevention: Economic perspectives. J. Dent. Res..

[24] Thomson W.M., Williams S.M., Broadbent J.M., Poulton R., Locker D. (2010). Long-term dental visiting patterns and adult oral health. J. Dent. Res..

[25] Guarnizo-Herreño C.C., Watt R.G., Fuller E., Steele J.G., Shen J., Morris S., Wildman J., Tsakos G. (2014). Socioeconomic position and subjective oral health: Findings for the adult population in England, Wales and Northern Ireland. BMC Public Health.

[26] Enabulele J., Chukwumah N.M. (2015). Socio-demographic determinants of utilization of oral health services among secondary school students. J. Oral Health Community Dent..

[27] Palència L., Espelt A., Cornejo-Ovalle M., Borrell C. (2014). Socioeconomic inequalities in dental care use. Community Dent. Oral Epidemiol..

[28] Eurostat Unmet Needs for Medical and Dental Care Statistics. https://ec.europa.eu/eurostat.

[29] Organisation for Economic Co-Operation and Development (OECD) OECD Health Statistics 2024. https://www.oecd.org.

[30] World Health Organization Global Health Observatory Data Repository. https://www.who.int/data/gho.

[31] OECD, European Union (2022). Health at a Glance: Europe 2022.

[32] OECD, European Union (2024). Health at a Glance: Europe 2024.

[33] Thomson S., Cylus J., Evetovits T., Jakab M., Murauskiene L., Karanikolos M. (2024). Financial protection in Europe. Lancet Reg. Health Eur..

[34] Elani H.W., Harper S., Allison P.J., Bedos C., Kaufman J.S. (2012). Socio-economic inequalities and oral health in Canada and the United States. J. Dent. Res..

[35] Winkelmann J., Rossi J.G., Schwendicke F., Dimova A., Atanasova E., Habicht T. (2022). Variation in dental care coverage and access across European countries. BMC Oral Health.

[36] Watt R.G., Daly B., Allison P., Macpherson L.M.D. (2020). Prevention and inequalities in oral health. J. Dent. Res..

[37] Choi S.E., Simon L., Riedy C.A. (2023). Impact of COVID-19 on dental care utilization patterns. Int. J. Environ. Res. Public Health.

[38] Kranz A.M., Chen A., Gahlon G., Stein B.D. (2021). 2020 trends in dental office visits during the COVID-19 pandemic. J. Am. Dent. Assoc..

[39] Henschke C., Winkelmann J., Eriksen A. (2023). Oral health coverage and access to care in Europe. Health Policy.

[40] Perpelea A.-C., Sfeatcu R., Tușaliu M., Tănase M., Meleșcanu Imre M., Ripszky Totan A., Funieru C., Nicolescu D.N., Pițuru S.-M. (2024). Exploring the Threefold Viewpoint on Children’s Oral Health in a Cross-Sectional Study. Healthcare.

[41] Perpelea A.-C., Sfeatcu R., Pițuru S.-M., Furtunescu F.L. (2026). Inequity in Schoolchildren’s Access to Oral Health Services in Romania: Implications for Public Oral Health Policies. Healthcare.

[42] World Health Organization (2024). Global Strategy and Action Plan on Oral Health 2023–2030.

